# Comparison of two self-reported measures of physical work demands in hospital personnel: A cross-sectional study

**DOI:** 10.1186/1471-2474-9-61

**Published:** 2008-04-29

**Authors:** Kirsten Nabe-Nielsen, Nils Fallentin, Karl B Christensen, Jette N Jensen, Finn Diderichsen

**Affiliations:** 1 The National Research Centre for the Working Environment, Copenhagen, Denmark; 2 Department of Social Medicine, Institute of Public Health, University of Copenhagen, Denmark

## Abstract

**Background:**

Low back pain (LBP) is a frequent health complaint among health care personnel. Several work tasks and working postures are associated with an increased risk of LBP. The aim of this study was to compare two self-reported measures of physical demands and their association with LBP (the daily number of patient handling tasks and Hollmann's physical load index).

**Methods:**

A questionnaire was distributed to 535 hospital employees in a psychiatric and an orthopedic ward in a Danish hospital. Of these 411 (77%) filled in and returned the questionnaire. Only the 373 respondents who had non-missing values on both measures of physical demands were included in the analyses. The distribution of physical demands in different job groups and wards are presented, variance analysis models are employed, and logistic regression analysis is used to analyze the association between measures of physical demands and LBP.

**Results:**

In combination, hospital ward and job category explained 56.6% and 23.3% of the variance in the self-reported physical demands measured as the daily number of patient handling tasks and as the score on the physical load index, respectively. When comparing the 6% with the highest exposure the prevalence odds ratio (POR) for LBP was 5.38 (95% CI 2.03–14.29) in the group performing more than 10 patient handling tasks per day and 2.29 (95% CI 0.93–5.66) in the group with the highest score on the physical load index.

**Conclusion:**

In specialized hospital wards the daily number of patient handling tasks seems to be a more feasible measure of exposure when assessing the risk of LBP compared to more advanced measures of physical load on the lower lumbar spine.

## Background

Musculoskeletal pain is a common health complaint in the general population and a significant part of all musculoskeletal pain is related to unspecific low back pain (LBP). It is estimated that 44–54% of the 30–50 year old Nordic population have experienced back pain at least once during a one-year period [1]. It is generally found that LBP is more frequent among nursing personnel compared to many other occupational groups [2-5].

Several physical exposures in the working environment have been linked to an increased risk of LBP, and a number of these are present in the working environment of hospital personnel. In a report by the National Research Council and Institute of Medicine (US) it is concluded that there is a clear relationship between back disorders and physical load imposed by lifting and/or carrying loads, frequent bending and twisting, physically heavy work, and whole-body vibration [3].

The majority of studies in this area are based on self-reported questionnaire data. Despite methodological flaws, self-reported exposure measurement has several advantages compared to technical exposure measurement and laboratory studies [6]. The feasibility of questionnaire-based instruments is generally high in epidemiological studies and several instruments have been developed in order to measure the perceived physical demands in the working environment [7-10]. These instruments can serve to unveil risk factors for negative health outcomes and to identify high risk jobs and work tasks.

The physical demands in the hospital sector can be measured with a generic questionnaire including several general questions on the relative frequency of different working postures and carrying of loads or by means of a single question related to the frequency of one specific but complex work tasks, e.g. the number of patient handling task during a normal work day.

Since both generic questionnaires with several items as well as specific questions are widely used, the overall aim of this study was to compare these two types of measures in a group of hospital personnel. First, we wanted to compare the accordance between the two types of self-reported exposure measurements and more objective measures of exposure as type of hospital ward and job category. Secondly, we wanted to investigate and compare the associations between each of the two types of self-reported exposure measurements and the occurrence of LBP.

The instrument based on several items was expected to imply less non-differential misclassification than a single item. In general, *non*-differential misclassification would lead to a bias toward the null value [11]. Compared to subjective appraisal of the relative frequency of work postures, asking specifically about the number of patient handling tasks was expected to lead to less differential misclassification. This may also yield smaller risk estimate. Therefore, we hypothesized that the association between LBP and a generic questionnaire with several items including subjective appraisals would be stronger than the association between LBP and a specific question on number of patient handling tasks.

## Methods

### Study population

A questionnaire based cross sectional study was carried out among hospital staff in the orthopedic and psychiatric ward in a Copenhagen hospital. These two wards are included in the study because of their preponderance of working environment problems. Permanently employed personnel engaged in nursing, treatment or counseling of patients were included in the study. Personnel on sick leave or maternity leave or employed in secretarial posts were excluded. A total of 535 employees were eligible for inclusion and were mailed a questionnaire. Of these 411 (77%) filled in and returned the questionnaire. The gender and age distribution did not differ significantly between respondents and non-respondents (data not shown).

Only 373 respondents who had non-missing values on both measures of physical demands were included in the analyses. The study population consisted of registered nurses (n = 128); other nursing staff (n = 114); physicians and psychologist (n = 75); physio- and ergotherapists (n = 33); and other hospital personnel (n = 23) i.e. social counselors, hospital orderlies, therapists and other clinical personnel. The distribution of personal characteristics and seniority in the study population is shown in Table 1.

**Table 1:** Personal characteristics and working experience among hospital personnel.

	**Included in the analyses (n = 373)**
**Gender**	
Women	79.6%
Men	20.4%
**Age **(mean/SD)	40.3 (10.5) yrs
**Ward**	
Orthopedic	32.4%
Psychiatric	67.6%
**Job title**	
Registered nurses	34.3%
Other nursing staff	30.6%
Physicians/psychologists	20.1%
Physio- and ergotherapist	8.8%
Other	6.2%
**Working hours per week **(mean/SD)	37.8 (6.8) hours
**Seniority in the present ward **(mean/SD)	3.6 (3.2) yrs
**Seniority in the health care system **(mean/SD)	11.6 (9.4) yrs

### Questionnaire

The questionnaire contained questions on work-related physical and psychosocial demands, musculoskeletal pain, individual characteristics and lifestyle factors.

#### Low back pain (LBP)

The questions on LBP were derived from the Standardised Nordic Questionnaire for the Analysis of Musculoskeletal Symptoms [12]. In this study the one-year prevalence of LBP was used as outcome. Cases were defined as participants with pain sometimes, often or very often during the last 12 month, while non-cases were defined as participants who reported pain seldom or never during the same period. This case-definition has previously been used [13,14].

#### Work-related physical demands

As an example of specific, single-item exposure measurement, the participants were asked about the number of patient handling tasks during a normal workday categorized as never, seldom, 1–2 times per day, 3–10 times per day, and more than 10 times per day. Answers were subsequently divided into three categories: 0–2 times per day, 3–10 times per day and more than 10 times per day. The generic instrument used in this study was Hollmann's physical load index [7]. The index was calculated as the weighted sum of the scores of 15 items describing the frequency of different work positions combined with the lifting of light to heavy objects. The weight of each item depended on estimated compressive forces on the lower lumbar spine from the posture given by that item. A score between 0 and 56.2 was calculated for each participant. The physical load index was categorized in three groups to yield a variable with a marginal distribution similar to that of the question on daily patient handling tasks. This enables comparison of the two instruments.

#### Work-related psychosocial demands

Demands related to social interactions (i.e. emotional demands and demands for hiding emotions), influence at work, possibilities for development and social support from leaders and colleagues were assessed using the Copenhagen Psychosocial Questionnaire [15]. For each participant a score between 0 and 100 was calculated. For the two demand scales a high score equaled high demands and was therefore potentially negative. For the three latter variables a high score reflected a resource that was potentially positive. All measures of psychosocial exposures were treated as continuous variables in the statistical analyses.

#### Personal characteristics and lifestyle factors

The participants were asked about their age and gender, physical activity during leisure time and smoking. Age was analyzed as a continuous variable, while physical activity was dichotomized as less than 4 and more than 4 hours per week; smoking was included in the analysis using three categories: non-smokers, former smokers and present smokers.

### Statistical analysis

The interrelationship between the measures of physical and psychosocial demands was assessed by computing Spearman correlation coefficients.

The relative contribution of patient handling tasks to the calculated overall physical load was assessed by computing the fraction of the residual variance explained by patient handling tasks in a variance analysis model for the physical load index. Next, the relative contribution of job category and ward to the variation in the two measures of physical demands was assessed by computing the fraction of the residual variance explained by job category and ward in variance analysis models for the physical load index and patient handling tasks, respectively.

The association between work factors and one-year prevalence of LBP was analyzed by logistic regression using SPSS (version 15.0). The protocol for analysis consisted of two steps. First, all the independent variables were analyzed in a univariable regression model with LBP as the dependent variable. Second, the adjusted association between LBP and the daily number of patient handling tasks or physical load index, respectively, was analyzed in at multivariable regression model. All other variables which were significantly associated with the outcome in the univariable analyses on a 5%-level were controlled for in the adjusted analyses.

## Results

The one-year prevalence of LBP was 39% in both nurses and other hospital personnel, 23% in physicians/psychologists, 33% in physio- and ergotherapists and 38% in other nursing staff.

Physical demands in the hospital sector as measured by a specific question on the daily number of patient handling tasks and the generic questions on working postures and lifting (the physical load index) are presented in Table 2 and 3. The Spearman correlation coefficient between the two measures of physical demands is 0.596, and the number of daily patient handling tasks explained 33.1% of the total variance in the physical load index. The Spearman correlation coefficient between the psychosocial demands and the two measures of physical demands ranged between -0.330 and 0.156, and the correlations between the five measures of psychosocial demands ranged between -0.355 and 0.364.

**Table 2:** Number of daily patient handling tasks in different job categories and wards; percentages are presented.

	**Orthopedic ward**				**Psychiatric ward**			
		
	**0–2**	**3–10**	**>10**	**n**	**0–2**	**3–10**	**>10**	**n**
Registered nurses	14.6%	70.8%	14.6%	48	96.3%	3.8%	-	80
Other nursing staff	-	54.5%	45.5%	33	92.6%	7.4%	-	81
Physicians/psychologists	93.8%	6.3%	-	32	100%	-	-	43
Physio- and ergotherapist	-	100%	-	2	100%	-	-	31
Other	33.3%	33.3%	33.3%	6	100%	-	-	17

**Table 3:** Physical load index score in different job categories and wards; percentages are presented.

	**Orthopedic ward**				**Psychiatric ward**			
		
	**Low** **(≤ 22.46)**	**Moderate** **(22.47–36.37)**	**High** **(≥ 36.38)**	**n**	**Low** **(≤ 22.46)**	**Moderate** **(22.47–36.37)**	**High** **(≥ 36.38)**	**n**
Registered nurses	39.6%	45.8%	14.6%	48	85.0%	13.8%	1.3%	80
Other nursing staff	30.3%	45.5%	24.2%	33	81.5%	13.6%	4.9%	81
Physicians/psychologists	81.3%	12.5%	6.3%	32	97.7%	2.3%	-	43
Physio- and ergotherapist	-	50.0%	50.0%	2	100%	-	-	31
Other	66.7%	16.7%	16.7%	6	94.1%	5.9%	-	17

Both types of exposure measurement of physical demands reflected the expected differences between job categories and at the same time distinguished between differences in physical demands between personnel within the same job category working in different wards. Of course, since the physical load index also quantifies physical demands apart from patient handling this instrument is preferable in job groups where the frequency of patient handling is low in order to be able to differentiate between groups as regards physical demands.

We found that, in combination, job category and ward explained 23.3% of the variance in the trichotomized physical load index and 56.3% of the variance in the trichotomized number of patient handling tasks. Thus, in this population patient handling tasks are more related to job title and ward than the physical load index. The residual variance not explained by ward and job category for each variable could be ascribed to e.g. individual factors, differences in patient-related factors and misclassification.

In the univariable analysis both the score on the physical load index as well as the daily number of patient handling tasks were significantly associated with the occurrence of LBP (Table 4). Also gender, age, demands for hiding emotions, influence at work, possibility for development, and social support are associated with LBP at a 5%-level and are therefore controlled for in the adjusted analyses.

**Table 4:** Crude prevalence odds ratios (POR) for low back pain, 95% confidence intervals and overall p-values.

	**POR (95% CI)**	**p-value**
**Patient handling tasks**		
0–2 per day	1.00	0.000
3–10 per day	2.29 (1.33–3.94)	
>10 per day	6.07 (2.43–15.20)	
**Physical load index**		
Low (≤ 22.46 points)	1.00	0.011
Middle (22.47–36.37 points)	2.10 (1.22–3.61)	
High (≥ 38.38 points)	2.29 (0.99–5.31)	
**Gender**		
Men	1.00	0.024
Women	1.94 (1.09–3.47)	
**Age**	1.02 (1.00–1.04)	0.039
**Leisure time physical activity**		
>4 hours per week	1.00	0.063
<4 hours per week	1.51 (0.98–2.35)	
**Smoking status**		
Non-smoker	1.00	0.533
Former smoker	1.26 (0.73–2.16)	
Present smoker	0.96 (0.56–1.64)	
Emotional demands*	1.12 (0.99–1.27)	0.071
Hiding emotions*	1.15 (1.02–1.30)	0.024
Influence at work*	0.87 (0.78–0.97)	0.015
Possibility for development*	0.85 (0.72–0.99)	0.042
Social support*	0.94 (0.89–0.99)	0.026

*) The effect of 10 points increase in exposure.

In the adjusted analyses the association between LBP and the number of daily patient handling tasks remained significant with POR's almost doubling between each exposure level, indicating a dose-response relationship between LBP and frequency of patient handling (Table 5). A similar association with LBP was also found for the physical load index although with a smaller increase in the POR between exposure levels.

**Table 5:** Adjusted* prevalence odds ratios for low back pain, 95% confidence intervals and p-values.

	**n (%)**	**POR (95% CI)**	**p-value**
**Patient handling tasks**			
0–2 per day	282 (75.6%)	1.00	0.000
3–10 per day	67 (18.0%)	2.98 (1.60–5.54)	
>10 per day	24 (6.4%)	5.38 (2.03–14.29)	
**Physical load index**			
Low (≤22.46 points)	282 (75.6%)	1.00	0.039
Middle (22.47–36.37 points)	67 (18.0%)	1.89 (1.04–3.44)	
High (≥ 36.38 points)	24 (6.4%)	2.29 (0.93–5.66)	

*) Gender, age, demands on hiding emotions, influence at work, possibility for development, and social support from leaders and colleagues.

These results demonstrate that both generic and specific measurements of physical demands in hospital work are significantly associated with an increased prevalence of LBP. However, when comparing the 6% of the population with the highest physical demands, as measured by each of the instruments, there is evidence that the relative risk of LBP when performing more than 10 daily patient handling tasks is higher than the risk in the high exposure group when using the physical load index. This is also supported by the p-values for each estimate.

## Discussion

In this cross-sectional study the one-year prevalence of LBP was associated with work-related physical demands among hospital personnel. The association was strongest when employing the daily number of patient handling task as a measure of exposure to physical demands. Although the physical load index is constructed as a measure of the added compressive forces on the lower lumbar spine during a normal work day this index turned out to be less exact in capturing the physical demands that increased the risk of reporting LBP. One reason could be that each patient handling situation implies a high risk of accidents due to sudden, unexpected loading. The physical load index, however, was a more sensitive measure of work-related physical demands in job groups and wards where the frequency of patient handling tasks was low.

Because of the high correlation between the two types variables measuring physical demands, the estimate for the association between patient handling tasks and LBP will also to some extent reflect the exposure to awkward postures and vice versa.

Several other studies have shown a relationship between patient-lifting frequency and low back problems [16]. According to Jensen, each patient handling involves an increased risk of a back injury especially when something unexpected happens (e.g. the patient slips) and in a meta-analysis of 6 epidemiologic studies the prevalence of low back problems among nursing personnel who frequently handled patients was 3.7 higher than the prevalence among personnel performing patient handling patients less frequently [17]. In a prospective study of nurses' aides the odds ratio for intense LBP was 1.63 when positioning patients in bed 5–9 times per day compared to zero times a day (the risk decreased when doing the same task 10 times or more per day) [14]. In another cross-sectional study, however, working for long periods with head, arms or body in awkward positions or working while bent or twisted at the waist were generally more strongly associated with back musculoskeletal disorders (OR 3.4–4.9) than physical demands which specifically involved patients (OR 2.0–2.8) [18].

Also psychosocial factors are found to be of importance, especially in relation to the course of LBP from an acute to a chronic state, and to the degree of disability caused by LBP [19-21]. The estimates of the association between physical demands and LBP have therefore been controlled for differences in psychosocial work factors. Including several job categories in the study population advantageously increased the contrast in exposure but did also enhance the risk of residual confounding. We did not control for job category in the adjusted analyses since job category is highly correlated with work-related demands and further adjustment would consequently weaken the study's ability to investigate the effect of different physical exposure measurements. Also the employment of (only) two wards increased the risk of residual confounding since we were not able to control for e.g. dimensions of work culture at the two wards.

The cross-sectional study design has limitations related to selection bias in terms of the healthy worker effect. This tends to yield conservative estimates of the association between physical demands and LBP. However, we expect this bias to equally influence the estimates for both measures of physical demands. Moreover, we can not determine causal relationships between physical demands and LBP in this study. On the other hand, this study can provide basis for decisions regarding exposure measurement in large scale follow-up studies on causal risk factors for LBP in hospital personnel.

Differential misclassification could be a source of bias in the present study, yielding spurious associations between exposure and outcome. Results of studies of validity and reliability of self-assessed physical demands point into different directions [2,10,22,23]. We assume that the number of patient handling tasks is a more "objective" measure which implies a lesser degree of individual interpretation than the frequency (in relative terms) of different working postures. We also found job category and ward to explain more of the variation in the daily patient handling than in the physical load index. Thus, the variable with the strongest association with LBP was also to a higher degree explained by more objective, though probably less precise, measures of exposure. These results indicate that differential misclassification between LBP cases and non-cases is not a major source of bias, even though differential misclassification can not unequivocally be ruled out.

## Conclusion

This study shows that among hospital personnel the frequency of patient handling tasks seems to be more strongly associated with LBP than a generic instrument estimating the total mechanical load on the lower lumbar spine. A single question on frequency of patient handling tasks therefore has advantages as a screening instrument both for practical reasons (e.g. the risk of missing data when asking several questions) and because of accuracy. It can be discussed whether these results can be generalized to other work places in the health care sector. At both the orthopedic and psychiatric ward the patients are highly selected with a relatively limited range of disabilities. It can be hypothesized that if the physical challenge involved in each patient handling task is even more diversified depending on the varying capacity and cooperation of each client the frequency of patient handling will be too unspecific as a measure of exposure.

## Competing interests

The authors declare that they have no competing interests.

## Authors' contributions

KNN participated in the formulation of the study, performed the statistical analyses and drafted the manuscript. NF participated in the formulation of the study and choice of statistical methods and was responsible for the design of the questionnaire survey and data collection. KBC participated in the formulation of the study, choice of statistical methods, and performance of statistical analyses. JNJ participated in the description of background knowledge. FD participated in the formulation of the study and choice of statistical methods, and had the overall scientific responsibility. All authors critically read, revised and finally approved the manuscript.

## Pre-publication history

The pre-publication history for this paper can be accessed here:


